# Integrated transcriptomic and tissue-validation analyses identify palmitoylation-associated biomarkers linked to sphingolipid metabolism and immune remodeling in psoriasis

**DOI:** 10.3389/fimmu.2026.1856647

**Published:** 2026-07-21

**Authors:** Qingqiong Luo, Rongcan Shi, Dandan Yang, Yijie Tang, Qinghui Xie, Yuling Shi, Fenyong Sun

**Affiliations:** 1 Department of Clinical Laboratory Medicine, Shanghai Skin Disease Hospital, School of Medicine, Tongji University, Shanghai, China; 2 Department of Dermatology, Shanghai Skin Disease Hospital, School of Medicine, Tongji University, Shanghai, China; 3 Institute of Psoriasis, School of Medicine, Tongji University, Shanghai, China; 4 Department of Clinical Laboratory Medicine, Shanghai Tenth People’s Hospital, School of Medicine, Tongji University, Shanghai, China

**Keywords:** immune microenvironment, keratinocytes, palmitoylation, psoriasis, sphingolipid metabolism, tissue validation

## Abstract

**Background:**

Psoriasis is a chronic immune-mediated inflammatory skin disease characterized by epidermal dysfunction, aberrant keratinocyte activation, and complex immune remodeling. Although lipid dysregulation has increasingly been implicated in psoriasis, the role of palmitoylation-associated genes in cutaneous inflammation remains insufficiently defined.

**Methods:**

We integrated bulk transcriptomic datasets (GSE14905, GSE13355, and GSE121212) and a single-cell RNA-sequencing dataset (GSE151177) to identify palmitoylation-associated biomarkers in psoriasis. Weighted gene co-expression network analysis, differential expression analysis, and machine-learning algorithms including LASSO and SVM-RFE were applied to screen hub genes. Functional enrichment, immune microenvironment analysis, regulatory-network construction, and candidate-drug prediction were subsequently performed. In addition, RT-qPCR was used to validate the expression of candidate genes in psoriatic lesional skin and healthy control skin.

**Results:**

We identified 209 palmitoylation-associated differentially expressed genes and screened five candidate hub genes: *CERK*, *SPTSSA*, *TNFSF10*, *CTNS*, and *HSD17B10*. External validation supported the relative robustness of *SPTSSA*, *TNFSF10*, CTNS, and *HSD17B10*, whereas *CERK* remained a biologically relevant mechanistic candidate. A four-gene score derived from *SPTSSA*, *TNFSF10*, CTNS, and *HSD17B10* defined a high-score state enriched in inflammatory, proliferative, and metabolic-stress pathways, including interferon responses, IL-6–JAK–STAT3 signaling, TNF signaling via NF-κB, complement, and inflammatory response. Immune deconvolution further revealed increased macrophage and activated dendritic-cell infiltration together with coordinated upregulation of multiple immune checkpoint molecules. Single-cell and correlation analyses suggested that *SPTSSA* showed relatively stronger expression in basal cells and keratinocytes, supporting a closer association with epidermal programs. *CTNS* also exhibited a mainly epidermal distribution, particularly in keratinocytes, whereas *TNFSF10* displayed a more immune-related pattern and *HSD17B10* showed a broader immune-metabolic distribution across multiple cell populations. RT-qPCR further supported the lesional upregulation of *SPTSSA* and *HSD17B10*, while *CERK* showed an opposite tissue-level trend and was therefore retained as a mechanistically informative candidate.

**Conclusion:**

This study identifies a palmitoylation-associated molecular signature related with sphingolipid metabolism, keratinocyte activation, and immune remodeling in psoriasis. These findings not only provide candidate biomarkers for disease stratification, but also highlight potentially druggable pathways and therapeutic targets for future translational investigation.

## 1 Introduction

Psoriasis is a chronic, relapsing, immune-mediated inflammatory skin disease that affects both cutaneous homeostasis and systemic health. Current pathogenic models emphasize the reciprocal interaction among activated keratinocytes, dendritic cells, macrophages, and pathogenic T-cell subsets, particularly within the IL-23/IL-17/TNF inflammatory circuit (1–3). However, psoriasis cannot be fully explained by cytokine dysregulation alone, because epidermal barrier abnormalities, metabolic reprogramming, and stress-response pathways are also tightly involved in disease maintenance (3–5).

Recent single-cell and immune-atlas studies have further refined this view by identifying disease-relevant myeloid populations and keratinocyte states in psoriatic lesions (6). In particular, inflammatory dendritic cells and type 3 dendritic-cell populations co-producing IL1B and IL23A appear to amplify local inflammatory circuits, while keratinocytes function not simply as passive targets but as active immune-regulatory cells that shape the lesional microenvironment (1, 7, 8). This framework supports the concept that psoriasis is a coordinated disorder of epidermal, stromal, and immune compartments rather than a purely lymphocyte-driven disease.

In parallel, increasing evidence indicates that sphingolipid metabolism is profoundly altered in psoriasis. Earlier biochemical studies reported reduced ceramide content, altered ceramide composition, and dysregulated expression of enzymes involved in ceramide biosynthesis in psoriatic skin (9–11). More recent work has shown lesion-specific ceramide remodeling, altered stratum-corneum ceramide profiles, and partial normalization of lipid abnormalities after effective biologic therapy (12–14). Experimental evidence further indicates that epidermal ceramide deficiency can directly drive psoriasis-like inflammation *in vivo (*15). Together, these observations suggest that lipid dysregulation is mechanistically important rather than merely epiphenomenal in psoriasis.

S-palmitoylation is a reversible lipid modification in which palmitate is covalently attached to target proteins, thereby influencing membrane localization, trafficking, stability, and signaling. Palmitoylation is increasingly recognized as a regulator of inflammatory and barrier-related processes in the skin, and zDHHC-mediated palmitoylation has been implicated in skin homeostasis and inflammatory signaling (16–18). In addition, aryl hydrocarbon receptor (AHR)-dependent signaling can influence sphingolipid programs, including SPTSSA-associated pathways, thereby providing a plausible mechanistic bridge between lipid metabolism, barrier regulation, and inflammatory skin disease (19–21).

Despite these advances, palmitoylation-associated biomarkers in psoriasis remain insufficiently characterized. Furthermore, recent machine-learning studies in psoriasis have identified several candidate biomarkers, but most were not framed within a palmitoylation- or sphingolipid-centered biological context (22–24). Therefore, in the present study, we integrated bulk transcriptomic datasets, single-cell data, machine-learning prioritization, immune microenvironment analysis, mechanism-oriented correlation analyses, and tissue-level RT-qPCR validation to identify palmitoylation-associated candidate biomarkers in psoriasis and to explore their connections with sphingolipid metabolism, epidermal activation, and inflammatory immune remodeling.

## 2 Materials and methods

### 2.1 Study design and data acquisition

**Figure 1:**
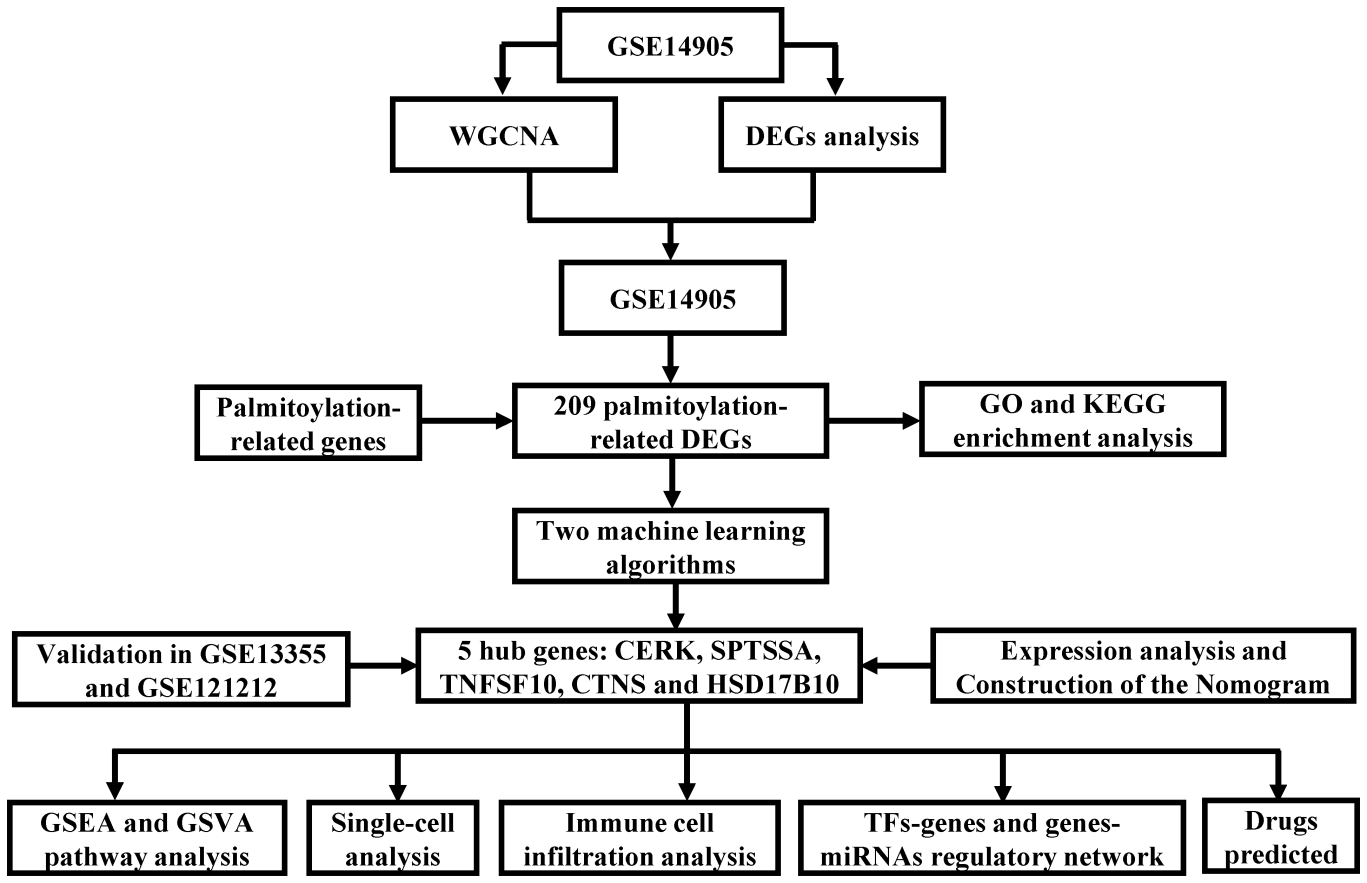
Study workflow chart.

A public-data-based integrative transcriptomic workflow was established to identify palmitoylation-associated candidate biomarkers in psoriasis (**Figure 1**). Three bulk transcriptomic datasets (GSE14905, GSE13355, and GSE121212) and one single-cell RNA-sequencing dataset (GSE151177) were obtained from the Gene Expression Omnibus (GEO, https://www.ncbi.nlm.nih.gov/geo/). GSE14905 was used as the training cohort for weighted gene co-expression network analysis (WGCNA), differential expression analysis, feature selection, and downstream signature construction. GSE13355 and GSE121212 served as external validation cohorts. GSE151177 was used for single-cell landscape analysis and cell-type-level expression profiling of prioritized genes. A total of 3,730 palmitoylation-related genes (PRGs) were retrieved from GeneCards (https://www.genecards.org/) using the search term “palmitoylation” (accessed on 2026-01-31). Duplicate entries were removed, and the resulting set was used as the broad association-level palmitoylation-related gene universe for initial screening (**Supplementary Table 1**). Because GeneCards integrates genes with varying degrees of direct and indirect relevance, this PRG list should be interpreted as a broad annotation resource rather than as a curated list of biochemically confirmed palmitoylated proteins.

### 2.2 WGCNA and identification of psoriasis-associated module genes

For weighted gene co-expression network analysis (WGCNA), the top 5,000 genes ranked by expression variability in GSE14905 were selected after preprocessing. A soft-thresholding power of 11 was chosen to satisfy the scale-free topology criterion. The weighted adjacency matrix was converted into a topological overlap matrix (TOM), and genes were hierarchically clustered using 1 - TOM as the dissimilarity measure. Modules were identified with dynamic tree cutting and merged when module eigengene (ME) similarity exceeded the prespecified threshold. The merging threshold is set at 0.25 (i.e., modules with a Pearson correlation coefficient greater than 0.75 between ME will be merged), and the minimum number of genes for each module is set at 70. Module–trait associations were then evaluated by correlating module eigengenes with psoriasis status. The module showing the strongest positive correlation with disease was retained for downstream analyses.

### 2.3 Differential expression analysis and identification of Palm-DEGs

Differential expression analysis between psoriasis and control samples in GSE14905 was performed using the “limma” R package after between-array normalization. Genes with adjusted *P* < 0.05 and |log_2_ fold change (FC)| > 0.585 were considered differentially expressed. To prioritize disease-activated candidates with greater translational plausibility as potential biomarkers or intervention-relevant targets, the initial screening focused on upregulated differentially expressed genes (DEGs). Specifically, upregulated DEGs were intersected with psoriasis-associated module genes and the GeneCards-derived PRG list. The resulting intersection set was operationally defined as palmitoylation-associated DEGs (Palm-DEGs). Because this strategy emphasized disease-upregulated candidates, biologically relevant downregulated genes were not included in the primary hub-gene screening framework.

### 2.4 Functional enrichment and score-based subgroup analysis

To characterize the biological context of the identified signature, a score was constructed from the prioritized genes and GSE14905 samples were stratified into high-score and low-score groups. Group-wise differential expression was analyzed with limma using adjusted *P* < 0.05 and |log_2_FC| > 0.585. Gene Ontology (GO) and Kyoto Encyclopedia of Genes and Genomes (KEGG) enrichment analyses were performed with clusterProfiler. Gene Set Enrichment Analysis (GSEA) was applied to ranked gene lists, and Gene Set Variation Analysis (GSVA) was performed using the Hallmark gene-set collection to compare pathway activities between score-defined subgroups.

### 2.5 Feature selection using machine learning

Palm-DEGs were subjected to two complementary feature-selection strategies: least absolute shrinkage and selection operator (LASSO) regression with 10-fold cross-validation and support vector machine-recursive feature elimination (SVM-RFE) with 5-fold cross-validation. Genes retained by both methods were considered candidate hub genes. Their expression patterns and receiver-operating-characteristic (ROC) performance were subsequently evaluated in the training and validation cohorts.

### 2.6 Immune microenvironment analysis

Immune-cell infiltration was evaluated in the training cohort using multiple deconvolution algorithms, including CIBERSORT, CIBERSORT-ABS, ESTIMATE, EPIC, IPS, MCPcounter, quanTIseq, TIMER, and xCell. Relative and absolute immune-cell abundance differences between score-defined groups were assessed. Co-stimulatory and co-inhibitory immune-regulatory molecules were profiled, key checkpoint genes were compared between groups, and Spearman correlation analysis was used to examine their relationships with the prioritized biomarkers.

### 2.7 Candidate-drug and regulatory-network analyses

Candidate compounds were predicted using the Comparative Toxicogenomics Database (CTD, http://ctdbase.org/). Chemicals associated with both psoriasis and prioritized hub genes were collected, manually filtered to remove environmental toxicants and low-relevance non-therapeutic compounds, and visualized as a disease–gene–drug network in Cytoscape. For regulatory-network analysis, skin-relevant transcription factors were obtained from hTFtarget (https://guolab.wchscu.cn/hTFtarget/#!/). Candidate miRNAs were predicted with miRWalk (http://mirwalk.umm.uni-heidelberg.de/) and retained only when supported by both TargetScan (https://www.targetscan.org/vert_80/) and miRDB (http://mirdb.org). A miRNA–gene–TF network was then constructed to identify upstream regulatory hubs.

### 2.8 Mechanism-oriented correlation analyses

Mechanism-oriented analyses were performed in GSE14905 by correlating the four most stable genes (*SPTSSA, TNFSF10, CTNS*, and *HSD17B10*) with representative marker genes for keratinocytes (KRT14, KRT5), fibroblasts (COL1A1, DCN), macrophages (C1QA, C1QC), dendritic cells (HLA-DRA, CD1C), and endothelial cells (PECAM1, CDH5). These genes were additionally correlated with representative pathways and markers related to IL-17/TNF inflammation, keratinization, myeloid inflammation, ceramide/sphingolipid metabolism, and AHR/xenobiotic responses. Only statistically significant Spearman correlations meeting the predefined display threshold (|r| ≥ 0.3, p < 0.05) are shown. Positive correlations are indicated in red and negative correlations in blue.

### 2.9 Single-cell RNA-sequencing analysis

Single-cell data from GSE151177 were processed with Seurat R package. Quality-control thresholds included 300–3,000 detected features, 1,000–20,000 counts, mitochondrial gene content < 5%, ribosomal gene content < 50%, and hemoglobin gene content < 0.1%. Highly variable genes were identified, principal-component analysis (PCA) was performed, Harmony was used for batch correction, and clustering was conducted using shared nearest-neighbor graph construction followed by uniform manifold approximation and projection (UMAP) visualization. Cell types were annotated according to established canonical markers. Finally, the expression abundance of prioritized genes was assessed across major cell populations in psoriasis and control samples.

### 2.10 Human skin samples and real-time PCR validation

To provide preliminary tissue-level validation, lesional skin tissues from five patients with psoriasis and healthy skin tissues from four control subjects were analyzed. Total RNA was extracted from skin tissues by TRIzol (Invitrogen, CA, USA), reverse-transcribed into cDNA with the PrimeScript RT Reagent kit (TaKaRa, Shiga, Japan), and subjected to quantitative PCR by the SYBR Premix Ex Taq II reaction system (TaKaRa) with gene-specific primers for *CERK*, *SPTSSA*, *TNFSF10*, *CTNS*, and *HSD17B10*. GAPDH was used as an endogenous control to normalize differences in the amount of total RNA of different samples. Primer sequences were listed in **Supplementary Table 2**. All procedures were approved by the Ethics Committee of Shanghai Skin Disease hospital (Approval No. 2020-36). Written informed consent was obtained according to institutional requirements. Each tissue sample was analyzed as one biological replicate, and RT-qPCR was performed in technical duplicate wells for each sample.

### 2.11 Statistical analysis

All statistical analyses were conducted primarily in R (version 4.4.1), with selected preprocessing steps in Python (version 3.10.18). Unless otherwise specified, two-group comparisons used Wilcoxon rank-sum tests and multiple-group comparisons used Kruskal–Wallis tests. Correlation analyses used Spearman coefficients. Tissue-level RT-qPCR comparisons between psoriatic lesions and healthy controls were analyzed using two-sided Mann–Whitney U tests. All statistical tests were two-sided, and *P* < 0.05 was considered statistically significant.

## 3 Results

### 3.1 WGCNA identified a psoriasis-associated co-expression module

**Figure 2:**
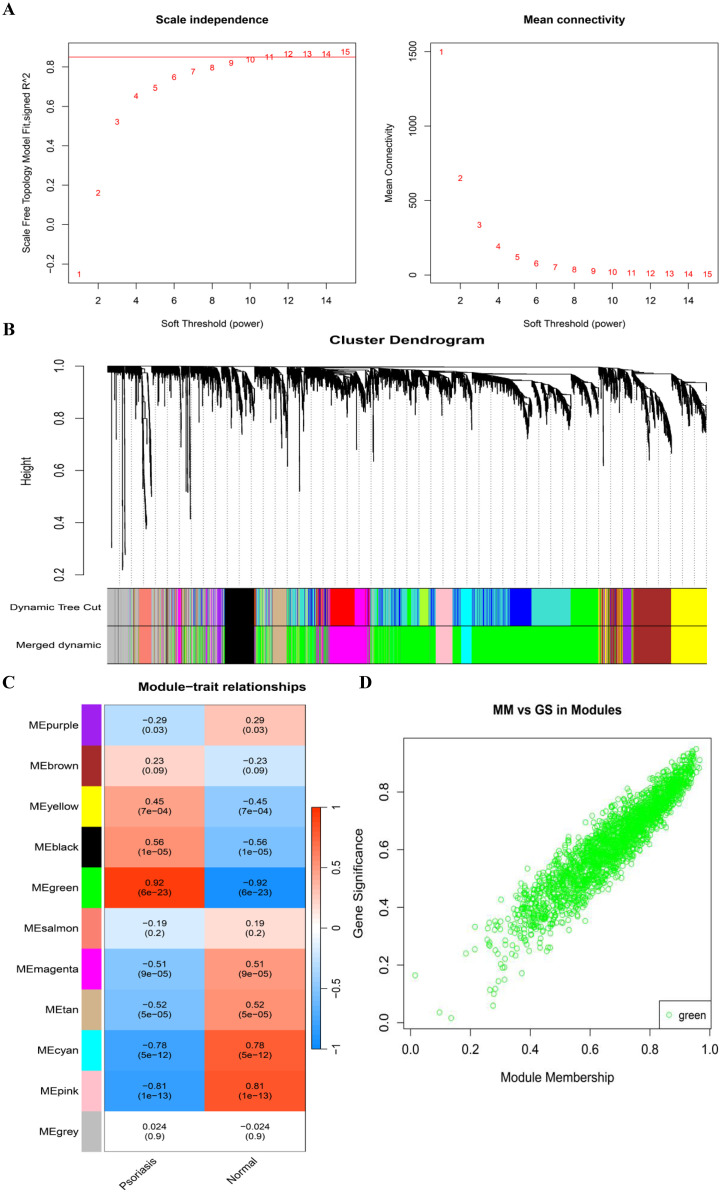
WGCNA identifies a psoriasis-associated co-expression module. **(A)** Scale-free topology analysis for soft-threshold selection. **(B)** Hierarchical clustering dendrogram with module assignment. **(C)** Heatmap of module–trait relationships. **(D)** Scatter plot showing the relationship between module membership and gene significance in the disease-associated module.

To identify gene co-expression modules associated with psoriasis, we performed WGCNA using the GSE14905 transcriptomic dataset. After preprocessing, the top 5,000 most variable genes were selected to minimize noise from low-variance genes. The pickSoftThreshold function was used to determine the optimal soft-thresholding power based on the scale-free topology fit index (R²) and mean connectivity. As shown in **Figure 2A**, a power of 11 achieved a relatively high scale-free topology fit (R² > 0.85) while maintaining acceptable connectivity, and was therefore chosen for network construction. A gene co-expression similarity matrix was then generated and transformed into a TOM. Based on TOM dissimilarity (1 - TOM), hierarchical clustering and dynamic tree cutting were performed to identify initial modules, followed by eigengene-based merging of highly similar modules (**Figure 2B**). In total, 11 stable co-expression modules were identified: black (n = 265), brown (n = 461), cyan (n = 108), green (n = 2,145), grey (n = 489), magenta (n = 472), pink (n = 243), purple (n = 160), salmon (n = 122), tan (n = 126), and yellow (n = 409). To assess module–trait relationships, Pearson correlation analysis was performed between module eigengenes and psoriasis status. The green module showed the strongest positive correlation with psoriasis (cor = 0.92, p = 6 × 10^-23^), suggesting that it may represent a key disease-associated module (**Figure 2C**). Notably, the same module showed a corresponding strong negative correlation with the normal-control trait (cor = −0.92, p = 6 × 10^-23^), further supporting its disease specificity. The cyan, pink, magenta, and tan modules also showed moderate correlations, although weaker than that of the green module. Further analysis of the green module demonstrated a strong positive correlation between module membership (MM) and gene significance (GS) (**Figure 2D**), indicating that highly connected genes within this module were also more strongly associated with the psoriatic phenotype. Together, these results support the green module as the key psoriasis-associated module for subsequent hub-gene screening.

### 3.2 Identification and functional characterization of palmitoylation-related differentially expressed genes

**Figure 3:**
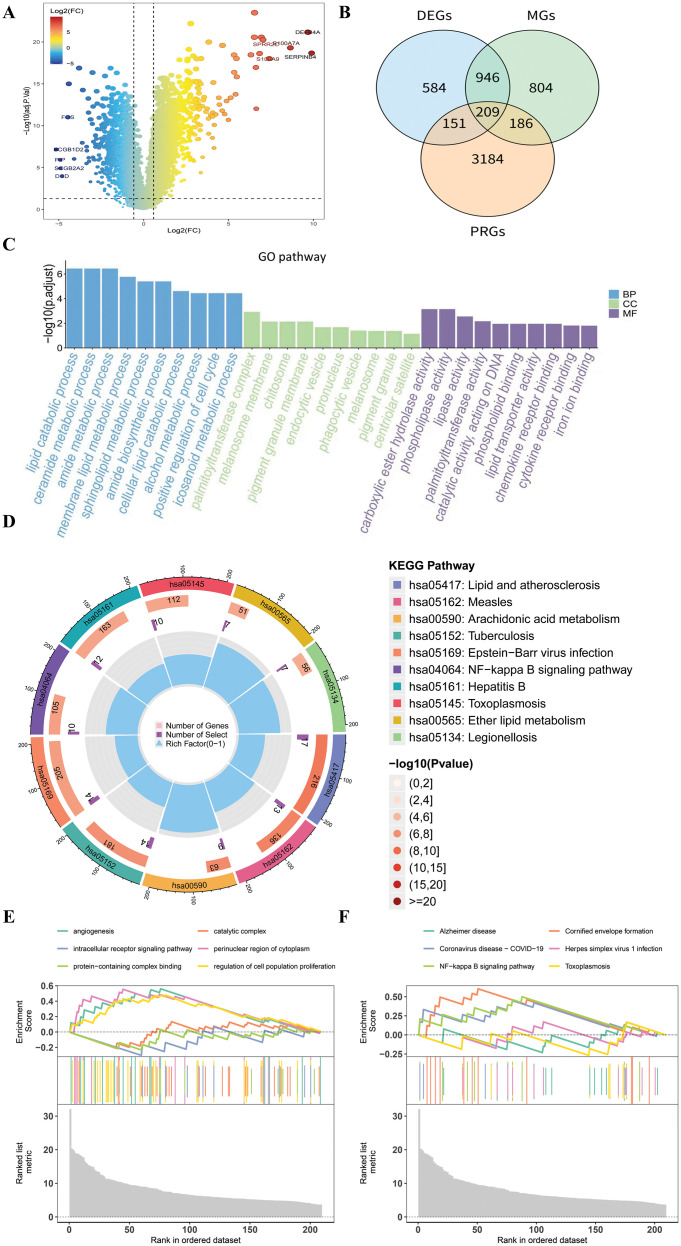
Identification of palmitoylation-associated differentially expressed genes. **(A)** Volcano plot of psoriasis versus control DEGs in GSE14905. **(B)** Venn diagram showing the intersection among upregulated DEGs, disease-associated module genes, and palmitoylation-related genes. **(C)** GO enrichment analysis of Palm-DEGs. **(D)** KEGG enrichment analysis of Palm-DEGs. **(E)** GO-based GSEA results. **(F)** KEGG-based GSEA results.

To characterize transcriptomic differences between psoriasis and normal skin, differential expression analysis was performed in the GSE14905 dataset using the limma package. With the cutoff set at |log2FC| > 0.585 and adjusted *P* < 0.05, a total of 3,877 DEGs were identified, including 1,890 upregulated and 1,987 downregulated genes (**Figure 3A**). The volcano plot showed a clear separation between upregulated and downregulated genes. To further identify PRGs associated with psoriasis, the upregulated DEGs, WGCNA-derived module genes (MGs), and the broad GeneCards-derived PRG list were intersected. This analysis yielded 209 overlapping genes, which were defined as Palm-DEGs (**Figure 3B**).

To explore the biological functions of these Palm-DEGs, GO, KEGG, and GSEA analyses were subsequently performed. In the GO enrichment analysis, Palm-DEGs were mainly enriched in lipid metabolism-related biological processes, including ceramide metabolic process, membrane lipid metabolic process, and sphingolipid metabolic process, as well as terms related to cellular response to stimulus and regulation of cellular process (**Figure 3C**). In the cellular component (CC) category, these genes were mainly enriched in membrane, organelle membrane, and endoplasmic reticulum, whereas in the molecular function (MF) category they were associated with oxidoreductase activity, hydrolase activity, and protein binding. KEGG pathway analysis further showed that Palm-DEGs were significantly enriched in several metabolic and inflammatory pathways, including Lipid and atherosclerosis, Arachidonic acid metabolism, Ether lipid metabolism, and the NF-kappa B signaling pathway (**Figure 3D**), suggesting their involvement in both metabolic regulation and signal transduction. To further assess the global functional distribution of these genes, GSEA was performed using ranked gene lists. GO-GSEA showed enrichment in pathways related to regulation of cell population proliferation, angiogenesis, and intracellular receptor signaling pathway (**Figure 3E**). Consistently, KEGG-GSEA identified significant enrichment of pathways such as NF-κB signaling pathway, Coronavirus disease - COVID-19, and Cornified envelope formation (**Figure 3F**).

Taken together, these results indicate that Palm-DEGs are mainly involved in lipid metabolic processes, membrane-associated structures, and multiple signaling pathways, providing a functional basis for subsequent hub-gene screening.

### 3.3 Machine learning prioritized five candidate hub genes

**Figure 4:**
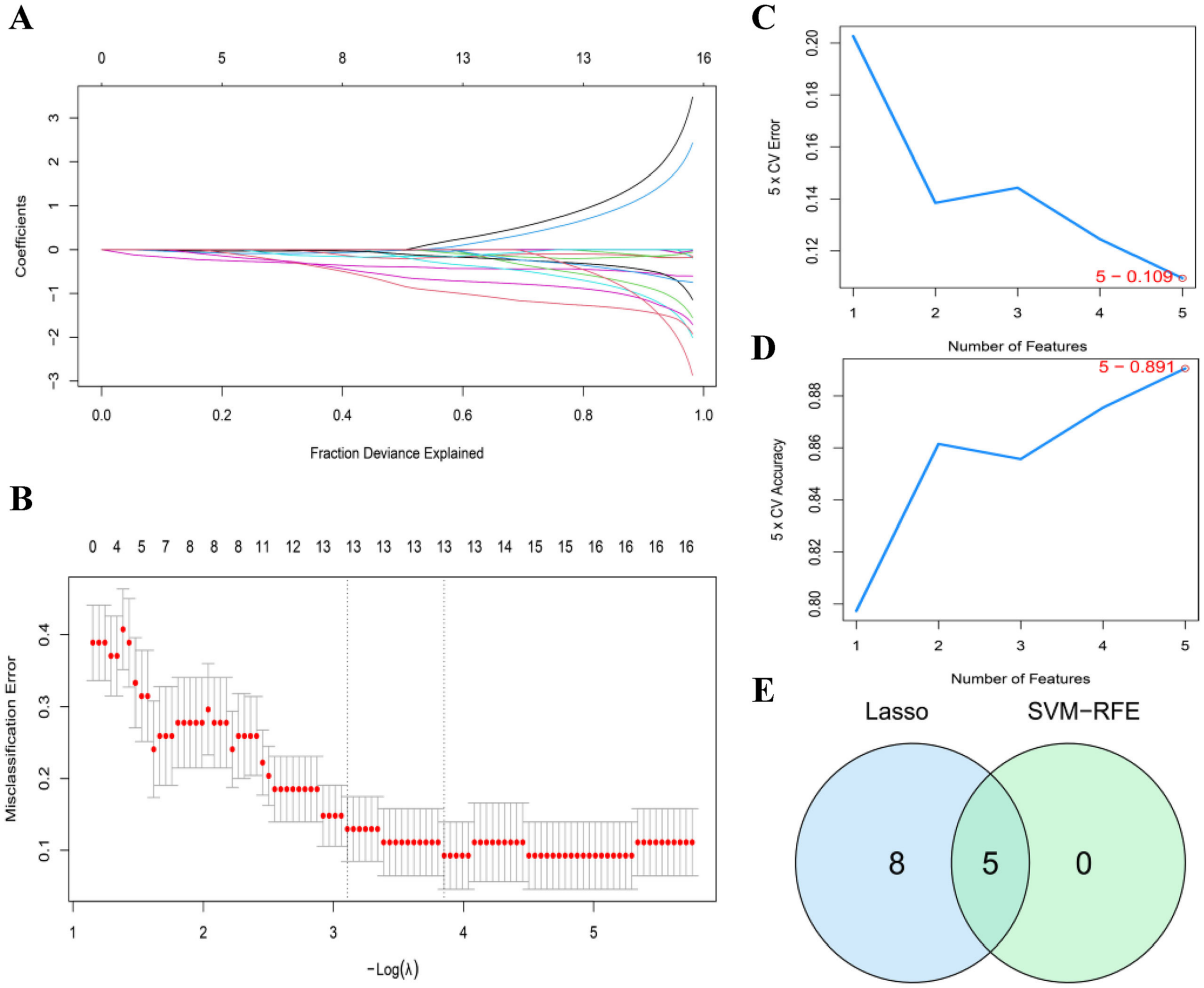
Machine-learning prioritization of hub genes. **(A)** LASSO coefficient profiles. **(B)** LASSO cross-validation curve. **(C)** SVM-RFE feature-selection process. **(D)** SVM-RFE model-accuracy curve. **(E)** Intersection of genes retained by LASSO and SVM-RFE.

Based on the 209 Palm-DEGs identified above, machine learning algorithms were applied to further screen candidate hub genes. Specifically, LASSO regression and SVM-RFE were used for feature selection. In the LASSO analysis, a penalized regression model with 10-fold cross-validation was constructed to determine the optimal λ value. At the optimal penalty parameter (lambda.min), 13 candidate genes were retained (**Figures 4A, B**), indicating that LASSO effectively reduced the dimensionality of the dataset while preserving informative features. In parallel, SVM-RFE was performed with 5-fold cross-validation to iteratively identify the most discriminative feature set. As shown in **Figures 4C, D**, model performance reached the optimum when the number of features was reduced to five, at which point the classification error was minimized (0.109) and the accuracy was maximized (0.891). To improve the robustness of hub-gene selection, the overlapping genes identified by LASSO and SVM-RFE were retained. This intersection analysis yielded five common candidate hub genes: *CERK*, *SPTSSA*, *TNFSF10*, *CTNS*, and *HSD17B10* (**Figure 4E**). These results indicate that the combined machine learning strategy effectively narrowed the Palm-DEG set to a small group of candidate hub genes for subsequent expression validation and diagnostic evaluation.

### 3.4 Expression patterns and diagnostic performance of the candidate hub genes were validated across independent datasets

To further validate the expression characteristics and diagnostic value of the identified hub genes, we examined their expression patterns and performed ROC curve analysis in the training cohort (GSE14905) and two independent validation cohorts (GSE13355 and GSE121212).

**Figure 5:**
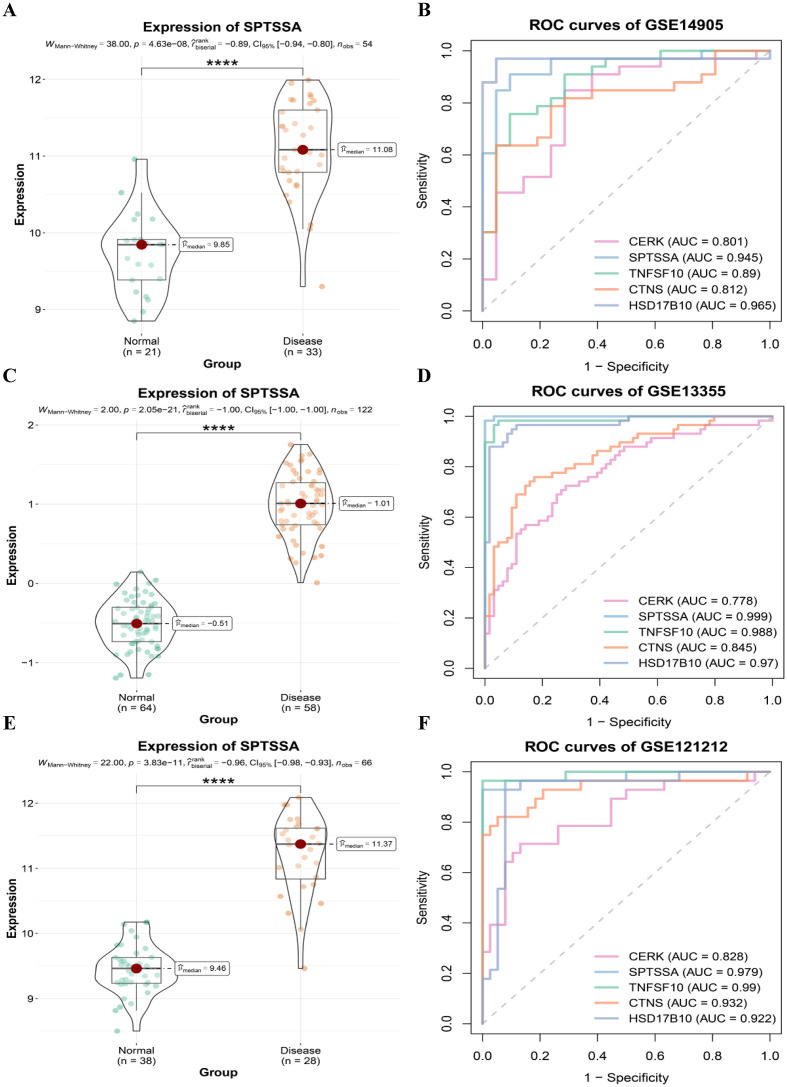
Cross-cohort validation of candidate hub genes. **(A)** Differential expression of candidate genes in GSE14905. **(B)** ROC curves in GSE14905. **(C)** Differential expression in GSE13355. **(D)** ROC curves in GSE13355. **(E)** Differential expression in GSE121212. **(F)** ROC curves in GSE121212. ****P < 0.0001.

In the training cohort GSE14905, the candidate hub genes showed differential expression between psoriatic and normal samples, with *SPTSSA* as a representative example showing significantly increased expression in the psoriasis group (**Figure 5A**). ROC analysis demonstrated that all five genes had certain discriminatory ability (**Figure 5B**). Among them, *SPTSSA* (AUC = 0.945), *TNFSF10* (AUC = 0.890), *CTNS* (AUC = 0.812), and *HSD17B10* (AUC = 0.965) showed relatively strong diagnostic performance, whereas *CERK* exhibited comparatively lower accuracy (AUC = 0.801). Overall, multiple candidate genes displayed good discriminatory capacity in the training set. In the first validation cohort GSE13355, the expression trends of these genes were generally consistent with those observed in the training cohort, and SPTSSA remained significantly upregulated in psoriatic samples (**Figure 5C**). ROC analysis further confirmed the robustness of several candidate genes (**Figure 5D**), including *SPTSSA* (AUC = 0.999), *TNFSF10* (AUC = 0.988), *CTNS* (AUC = 0.845), and *HSD17B10* (AUC = 0.970), while *CERK* again showed relatively weaker performance (AUC = 0.778). These findings further support the stable discriminatory potential of the selected genes. In the second independent validation cohort GSE121212, the candidate genes similarly showed consistent expression differences between groups (**Figure 5E**). ROC analysis showed that *TNFSF10* (AUC = 0.990), *SPTSSA* (AUC = 0.979), *CTNS* (AUC = 0.932), and *HSD17B10* (AUC = 0.922) retained strong diagnostic performance, whereas *CERK* again showed relatively limited discriminatory ability (AUC = 0.828) (**Figure 5F**). Overall, the results were highly consistent across datasets.

Taken together, the combined training and validation analyses indicate that *SPTSSA*, *TNFSF10*, *CTNS*, and *HSD17B10* showed relatively stable expression differences and diagnostic performance across independent cohorts, suggesting that they may serve as robust candidate hub genes for psoriasis identification and subsequent investigation. The expression patterns of the remaining candidate genes in each dataset are provided in **Supplementary Figure 1**.

### 3.5 Functional enrichment analysis of the key-gene signature revealed an immune-inflammatory high-score state

To systematically evaluate the integrated expression pattern of the key genes, a single-sample gene set enrichment analysis (ssGSEA) score was calculated for each sample based on the four relatively stable hub genes (SPTSSA, TNFSF10, CTNS, and HSD17B10). Samples were then divided into high-score and low-score groups according to the median score (**Supplementary Table 3**).

**Figure 6:**
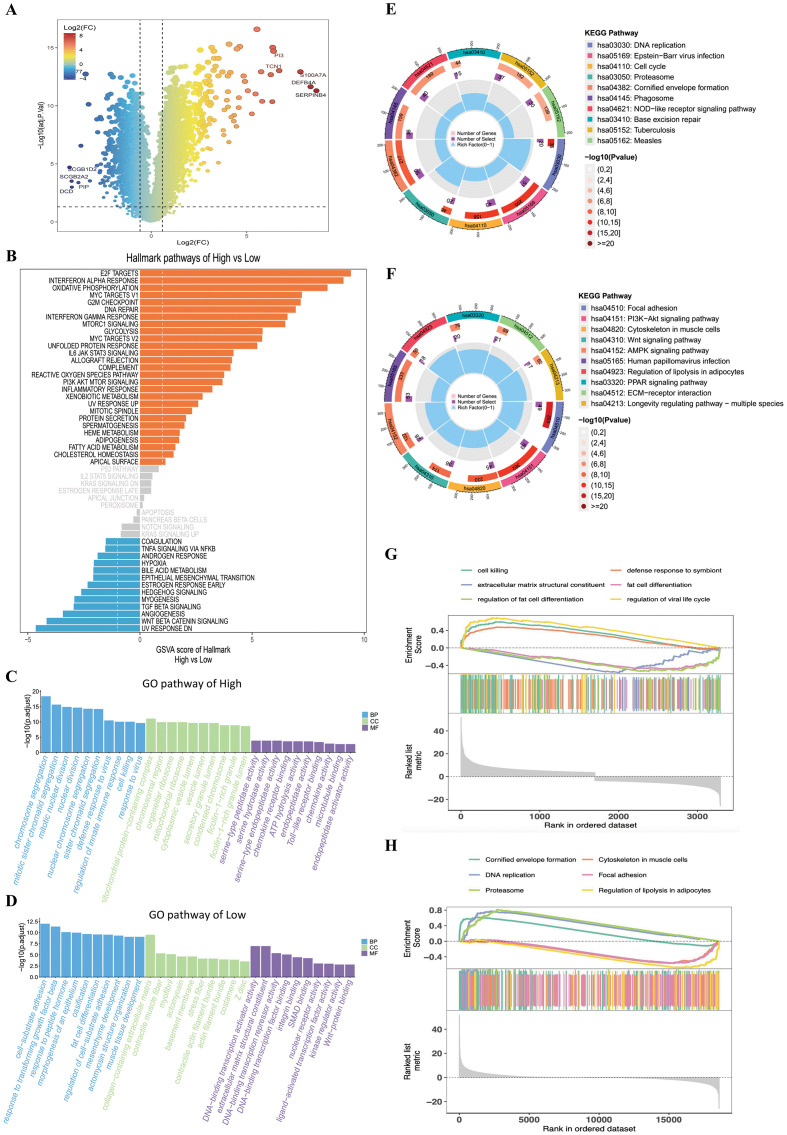
Functional enrichment analysis of score-defined subgroups. **(A)** Volcano plot of high-score versus low-score DEGs. **(B)** Hallmark GSVA pathway differences between score-defined groups. **(C, D)** GO enrichment of genes upregulated in the high-score and low-score groups, respectively. **(E, F)** KEGG enrichment of genes upregulated in the high-score and low-score groups, respectively. **(G, H)** GSEA results based on GO and KEGG gene sets.

Using GSE14905, differential expression analysis between the two groups identified 1,833 upregulated genes in the high-score group and 1,770 relatively upregulated genes in the low-score group (**Figure 6A**), indicating marked transcriptomic differences. GSVA showed that the high-score group was significantly enriched in pathways related to cell proliferation, immune activation, and metabolic stress, including E2F targets, G2M checkpoint, MYC targets, DNA repair, interferon alpha/gamma response, IL6-JAK-STAT3 signaling, TNFA signaling via NF-κB, complement, inflammatory response, oxidative phosphorylation, glycolysis, reactive oxygen species pathway, and unfolded protein response. In contrast, the low-score group was mainly enriched in Wnt/β-catenin signaling, TGF-beta signaling, Hedgehog signaling, angiogenesis, myogenesis, epithelial-mesenchymal transition and UV response DN (**Figure 6B**). Consistently, GO enrichment analysis showed that genes upregulated in the high-score group were mainly involved in cell cycle regulation, chromosome segregation, antiviral response, and immune-related signaling (**Figure 6C**), whereas genes enriched in the low-score group were associated with epidermal development, cell differentiation, response to TGF-beta, and extracellular matrix-related functions (**Figure 6D**). KEGG analysis further demonstrated that the high-score group was enriched in DNA replication, Cell cycle, Proteasome, Cornified envelope formation, NOD-like receptor signaling pathway, Base excision repair, Phagosome, Tuberculosis and Measles (**Figure 6E**). By contrast, the low-score group was mainly enriched in Focal adhesion, PI3K-Akt signaling pathway, Wnt signaling pathway, AMPK signaling pathway, ECM-receptor interaction, Human papillomavirus infection, Regulation of lipolysis in adipocytes, PPAR signaling pathway and Longevity regulating pathway - multiple species (**Figure 6F**). Finally, GSEA confirmed a global enrichment of immune response-related processes in the high-score group, together with pathways such as Cornified envelope formation, DNA replication, and Proteasome, whereas Focal adhesion and Regulation of lipolysis in adipocytes were enriched in the low-score direction (**Figures 6G, H**).

These findings indicate that the high-score group is characterized by enhanced immune-inflammatory activity, cell-cycle progression, and metabolic reprogramming, whereas the low-score group is more closely associated with tissue homeostasis and differentiation-related programs.

### 3.6 Immune microenvironment analysis revealed enhanced immune infiltration and checkpoint upregulation in the high-score group

**Figure 7:**
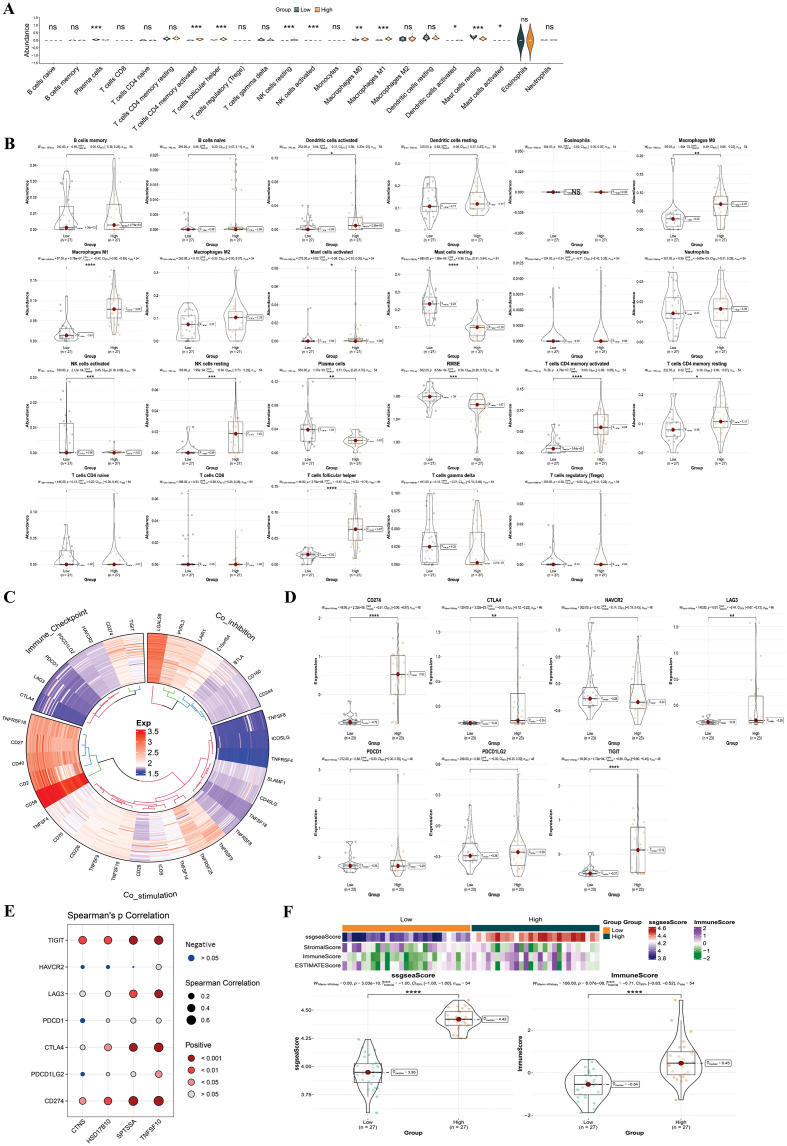
Immune microenvironment features associated with the palmitoylation-related score. **(A)** CIBERSORT-based differences in relative immune-cell abundance. **(B)** CIBERSORT-ABS-based differences in absolute immune-cell abundance. **(C)** Expression patterns of co-stimulatory and co-inhibitory immune-regulatory molecules. **(D)** Differential expression of key immune-checkpoint genes. **(E)** Correlation heatmap between stable biomarkers and immune-checkpoint molecules. **(F)** ESTIMATE-derived microenvironment scores. *P < 0.05, **P < 0.01, ***P < 0.001, ****P < 0.0001; ns, not significant.

To characterize immune microenvironment differences between the two score-defined groups, multiple immune deconvolution algorithms, including CIBERSORT, CIBERSORT-ABS, ESTIMATE, and MCPcounter etc., were applied to the GSE14905 dataset. Using CIBERSORT, the high-score group showed significantly increased infiltration of several immune cell subsets, including Macrophages M0, Macrophages M1, activated dendritic cells, activated CD4 memory T cells, T follicular helper cells, and plasma cells, whereas resting mast cells and activated NK cells were reduced (**Figure 7A**). This pattern suggests an immune microenvironment characterized by enhanced innate immune activation, antigen presentation, and adaptive immune engagement. Similar trends were confirmed by CIBERSORT-ABS, in which Macrophages M0/M1 and activated dendritic cells remained elevated, while resting mast cells were decreased in the high-score group (**Figure 7B**). Results from other deconvolution methods are provided in **Supplementary Figure 2**. We next assessed the expression of immune regulatory molecules. As shown in **Figure 7C**, both co-stimulatory and co-inhibitory molecules displayed an overall upward trend in the high-score group. Further analysis of representative immune checkpoint genes showed that CD274 (PD-L1), CTLA4, LAG3, and TIGIT were significantly upregulated, whereas PDCD1 and HAVCR2 showed no marked difference or only mild variation (**Figure 7D**). These findings indicate that immune activation in the high-score group is accompanied by concurrent induction of inhibitory immune signals. Finally, ESTIMATE analysis showed that the ImmuneScore was significantly higher in the high-score group, whereas the StromalScore and ESTIMATEScore showed no substantial changes (**Figure 7F**), indicating that the score model mainly reflects differences in immune-cell infiltration rather than stromal components. Taken together, these results indicate that the high-score group is characterized by enhanced immune infiltration, active inflammatory responses, and coordinated upregulation of immune checkpoint molecules, reflecting a complex immune state in which immune activation and immune inhibition coexist.

### 3.7 CTD-based therapeutic network analysis highlighted SPTSSA-associated candidate compounds and translational relevance

**Figure 8:**
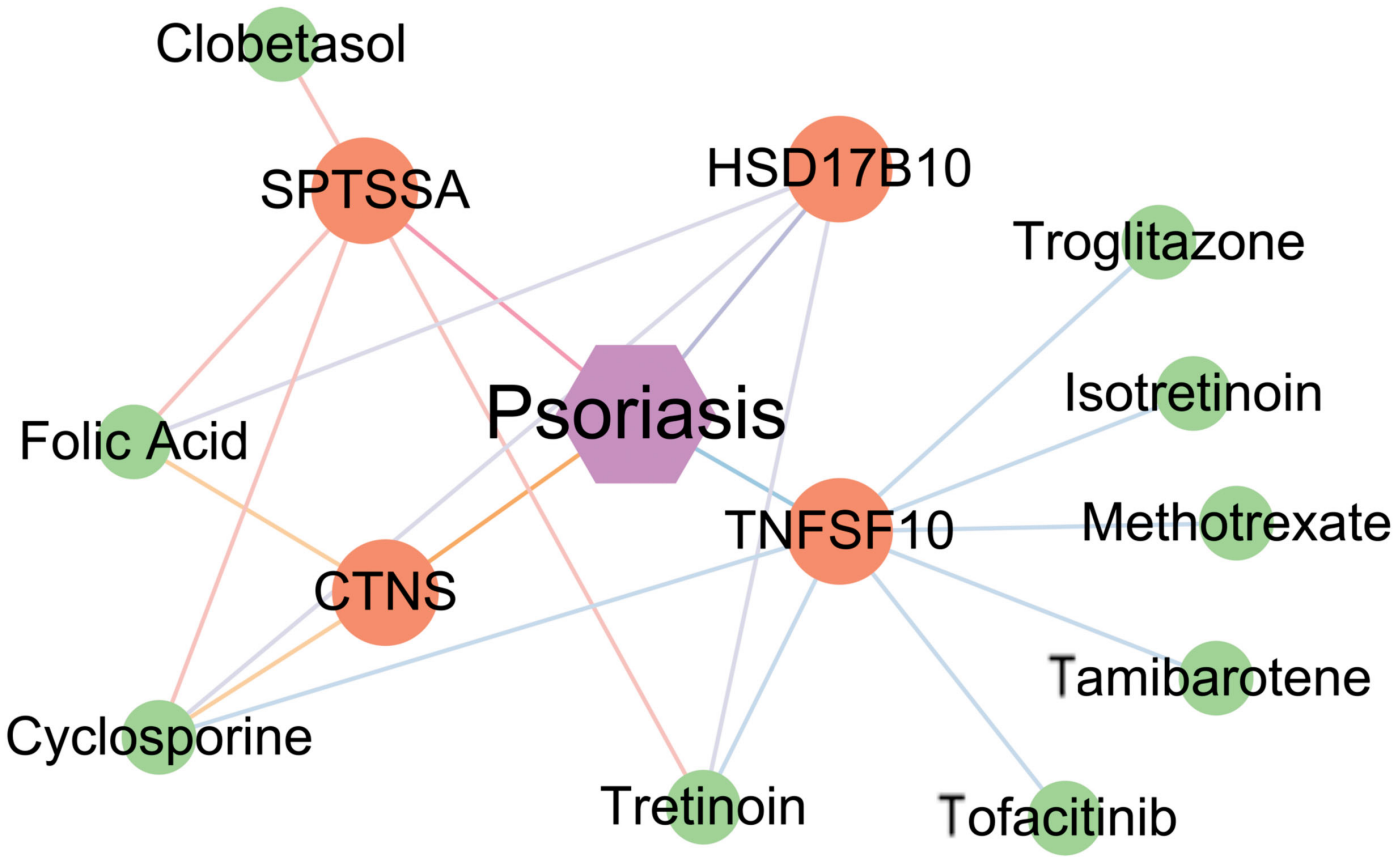
Therapeutic network analysis of SPTSSA-associated candidate compounds. Disease–gene–drug interaction network linking psoriasis, the stable biomarkers, and manually curated candidate compounds. Network centrality highlights SPTSSA as a prominent node.

To further explore the therapeutic relevance of the prioritized genes, a disease–gene–drug interaction network was constructed based on the CTD database (**Figure 8**). By integrating psoriasis-related genes and associated compounds, candidate drugs potentially linked to the identified hub genes were systematically screened. The resulting network included four key genes (*SPTSSA*, *TNFSF10*, *CTNS*, and *HSD17B10*) and multiple candidate compounds. Among them, *SPTSSA* was directly linked to several psoriasis-related compounds, suggesting that it may occupy a prominent position in the psoriasis-related therapeutic interaction network. Further analysis identified four candidate compounds associated with *SPTSSA*, namely Clobetasol, Cyclosporine, Tretinoin, and Folic Acid. Notably, Clobetasol and Cyclosporine are well-established therapeutic agents for psoriasis, primarily acting through anti-inflammatory and immunomodulatory mechanisms. Tretinoin, a retinoid derivative, is known to regulate keratinocyte proliferation and differentiation, whereas Folic Acid may participate in cellular metabolic regulation as an adjunctive factor. Importantly, these compounds are closely related to immune regulation, inflammatory responses, or keratinocyte function, which represent central pathogenic processes in psoriasis. These findings suggest that SPTSSA may be functionally involved in psoriasis-associated therapeutic networks and could represent a potential target with translational relevance.

### 3.8 Regulatory network analysis highlighted SPTSSA as a central node

**Figure 9:**
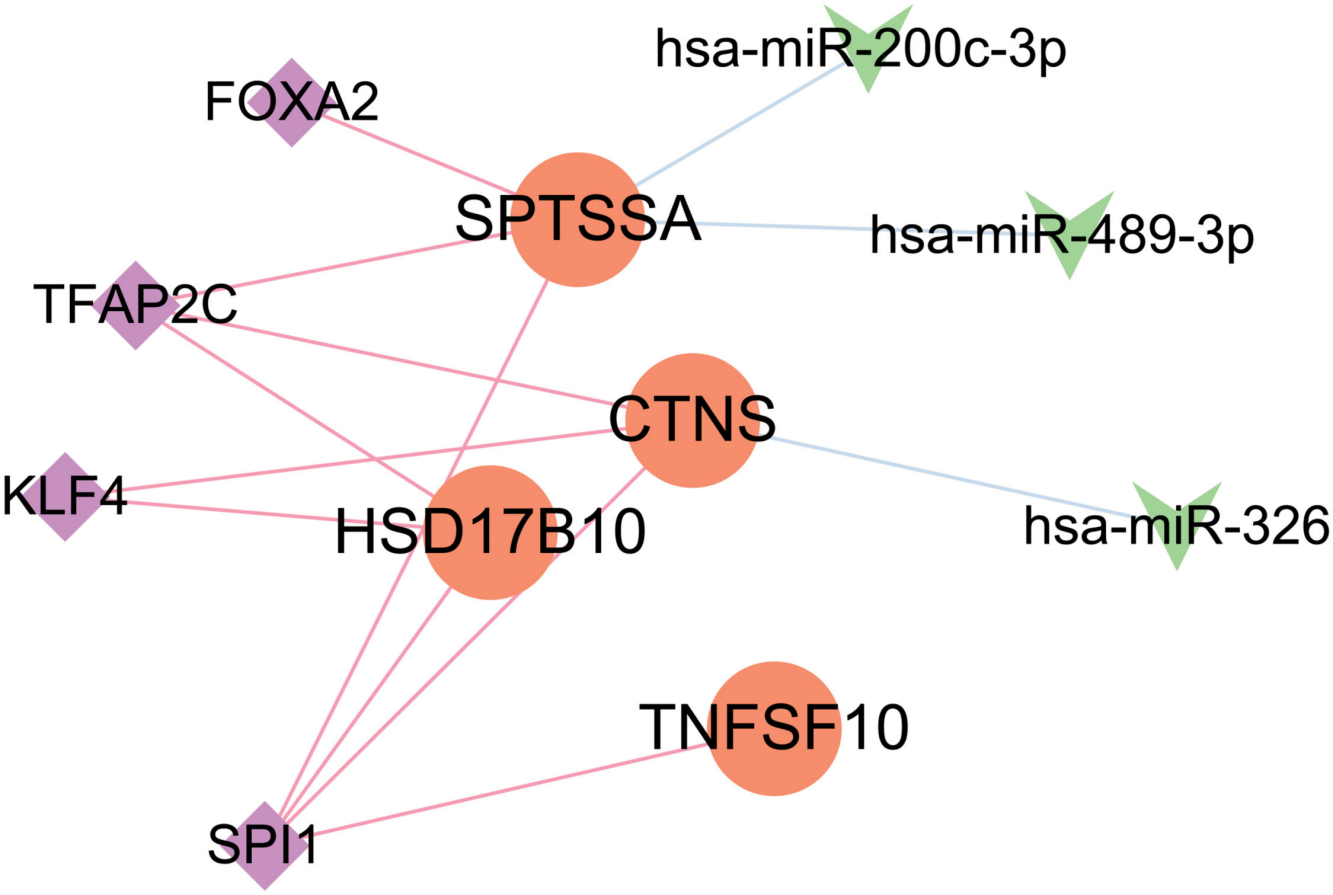
Integrated miRNA–gene–TF regulatory network. Skin-relevant transcription factors and high-confidence candidate miRNAs regulating the stable biomarkers are shown in a multilayer network, highlighting upstream regulatory convergence on *SPTSSA*.

To further explore the upstream regulatory mechanisms of the key genes, a miRNA–gene–TF regulatory network was constructed based on transcription factor and miRNA information. Skin-related transcription factors targeting the four key genes (*SPTSSA*, *TNFSF10*, *CTNS*, and *HSD17B10*) were obtained from the hTFtarget database, and candidate miRNAs were predicted using miRWalk, followed by filtering with TargetScan and miRDB to improve reliability. The integrated regulatory network is shown in **Figure 9**. Within this network, all four key genes occupied central positions and were connected to multiple transcription factors and miRNAs. At the transcription factor level, SPI1, TFAP2C, FOXA2, and KLF4 showed potential regulatory relationships with the key genes, suggesting their possible involvement in upstream transcriptional control. Among them, SPI1 was linked to multiple key genes and may represent an important immune-related regulatory node. At the miRNA level, several high-confidence miRNAs, including hsa-miR-200c-3p, hsa-miR-489-3p, and hsa-miR-326, were predicted to target the key genes, indicating that post-transcriptional regulation may also contribute to their expression control. Notably, *SPTSSA* showed the broadest range of predicted connections with both transcription factors and miRNAs, suggesting that it may act as a central regulatory node in this network. Taken together, these findings suggest that the identified key genes may be regulated through both transcription factor- and miRNA-mediated mechanisms, with *SPTSSA* occupying a particularly prominent position in the integrated regulatory network.

### 3.9 Mechanistic exploration suggested keratinocyte predominance and links to inflammatory and sphingolipid pathways

**Figure 10:**
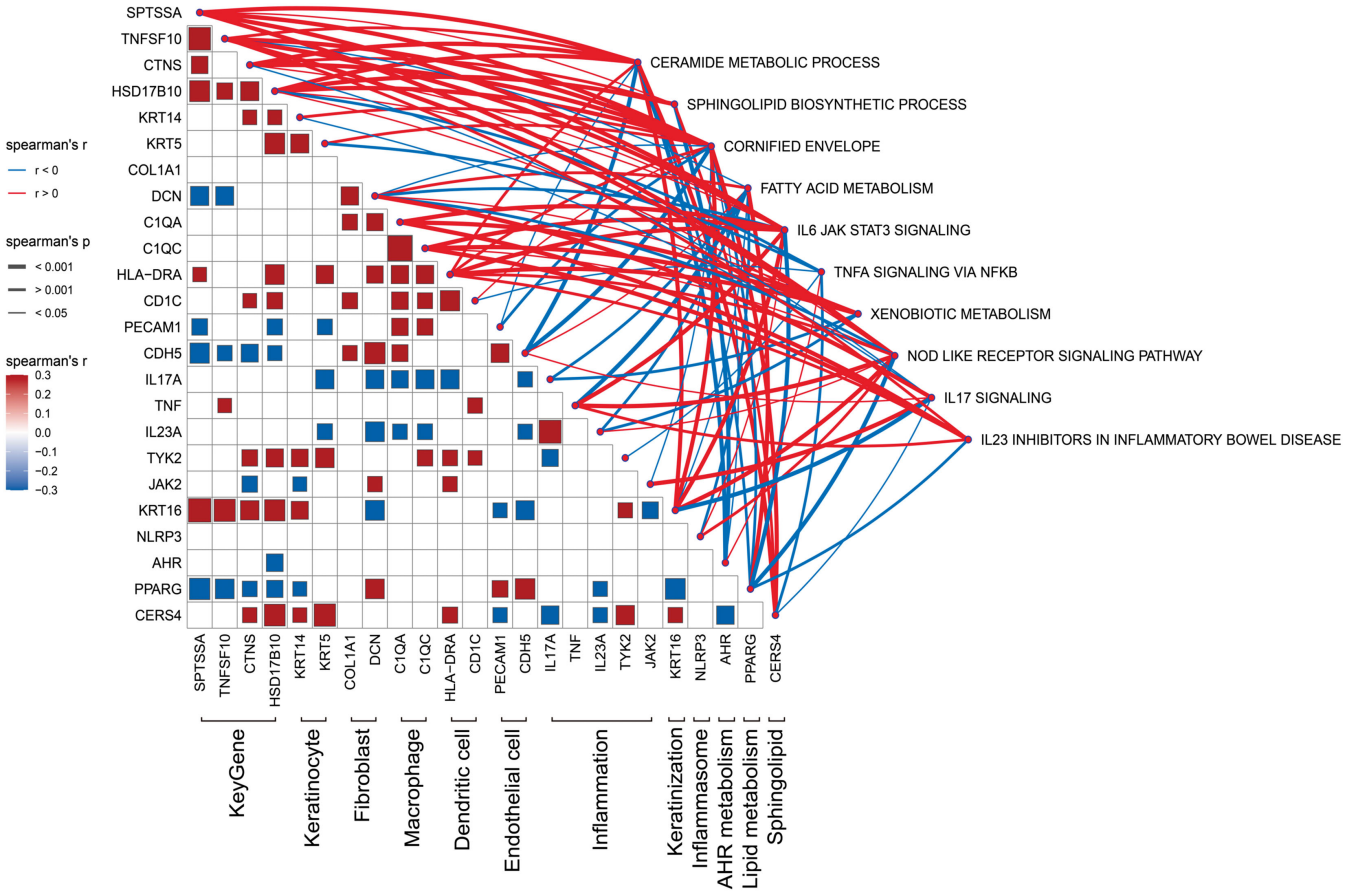
Thresholded correlation analysis of prioritized biomarkers and psoriasis-relevant cellular and pathway features. Correlation heatmap showing statistically significant Spearman associations of *SPTSSA*, *TNFSF10*, *CTNS*, and *HSD17B10* with marker genes of keratinocytes, fibroblasts, macrophages, dendritic cells, and endothelial cells, as well as psoriasis-relevant inflammatory and metabolic pathways. Blank cells indicate correlations below the predefined display threshold or non-significant associations.

To further investigate the potential roles of the key genes, thresholded Spearman correlation analyses were performed in GSE14905 between the four key genes (*SPTSSA*, *TNFSF10*, *CTNS*, and *HSD17B10*), representative cell-type marker genes, and psoriasis-related pathways (**Figure 10**). Only statistically significant correlations meeting the predefined display threshold are shown, thereby highlighting the most interpretable associations. Overall, the four genes showed distinct but partially overlapping correlation patterns. *SPTSSA* and *CTNS* displayed clearer positive correlations with epidermal and keratinization-related markers, particularly KRT14, KRT5, and KRT16, supporting a closer association with keratinocyte-lineage programs. By contrast, correlations with fibroblast and endothelial markers were generally weak or negative, arguing against a dominant stromal or endothelial origin. *TNFSF10* and *HSD17B10* showed relatively stronger positive correlations with immune-related markers, especially dendritic-cell and macrophage-associated markers, suggesting a closer relationship with inflammatory immune-cell programs.

At the pathway level, the four genes were linked to multiple psoriasis-relevant inflammatory and metabolic processes, but the dominant associations differed by gene. *SPTSSA* showed broad positive correlations with ceramide metabolic process, sphingolipid biosynthetic process, cornified envelope, and several inflammatory signaling pathways, including IL6–JAK–STAT3 signaling, TNFA signaling via NF-κB, and IL17 signaling, supporting a close association with epidermal differentiation, sphingolipid metabolism, and inflammatory activation. *CTNS* shared part of this epidermal/inflammatory correlation pattern, whereas *TNFSF10* and *HSD17B10* were more strongly connected to immune-inflammatory modules, including antigen-presentation- and cytokine-related programs. Associations with AHR/xenobiotic metabolism and selected lipid-metabolism-related signatures were also observed.

Taken together, these results suggest that the four prioritized genes occupy partially distinct biological contexts in psoriasis: *SPTSSA* and *CTNS* appear more closely related to epidermal and keratinization-associated programs, whereas *TNFSF10* and *HSD17B10* appear more strongly linked to immune-inflammatory remodeling. Among them, *SPTSSA* showed the broadest correlation pattern spanning keratinocyte-associated markers, sphingolipid metabolism, and inflammatory signaling, highlighting its role as a biologically prominent association-level candidate in psoriasis.

### 3.10 Single-cell landscape and cell-type-specific expression patterns of prioritized genes in psoriatic skin

**Figure 11:**
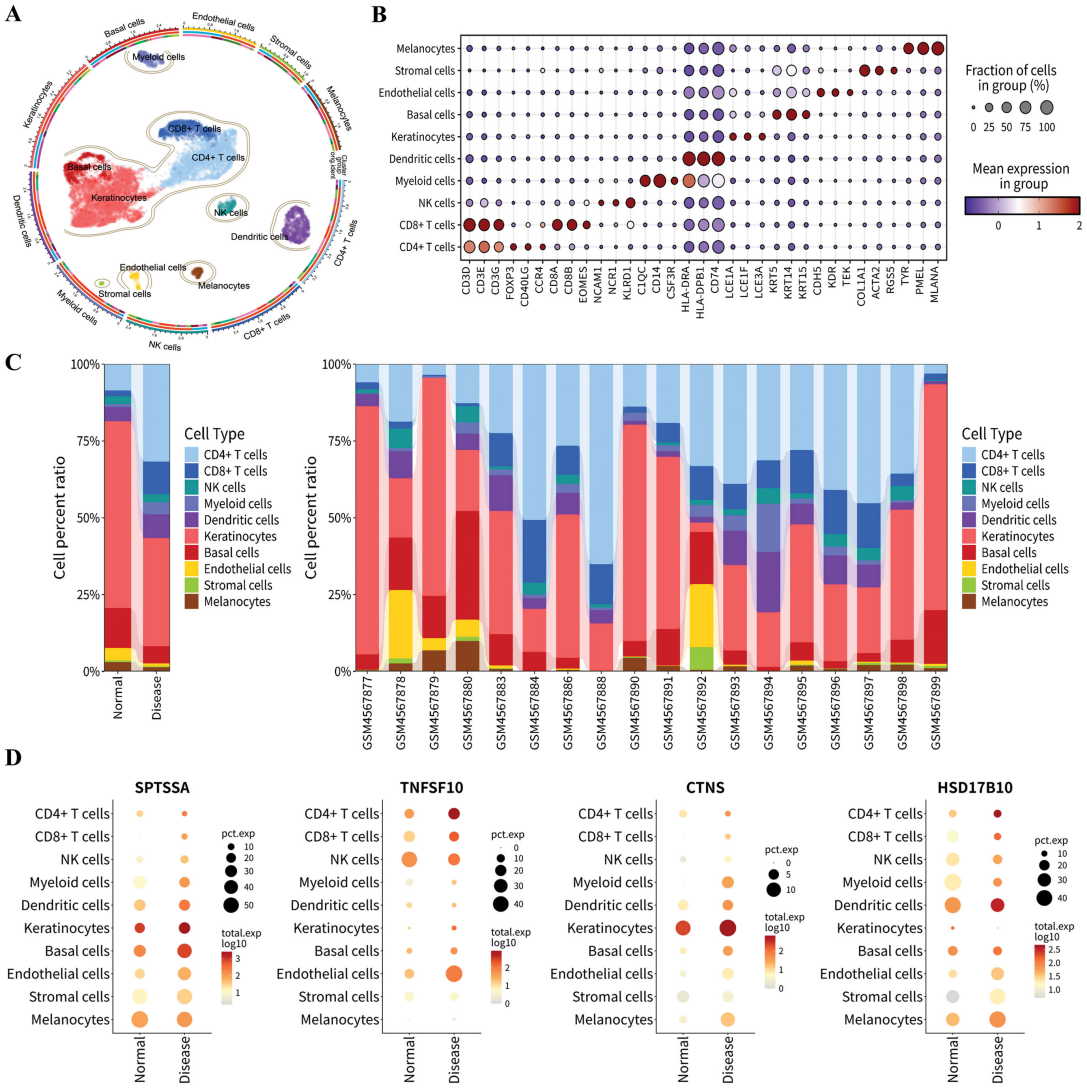
Single-cell landscape of psoriasis samples in GSE151177. **(A)** UMAP-based cell annotation. **(B)** Dot plot of canonical cell-type markers. **(C)** Relative cell-type proportions in psoriasis and control samples. **(D)** Dot plots showing the expression distributions of *SPTSSA*, *TNFSF10*, *CTNS*, and *HSD17B10* across major cell types in psoriasis and control samples.

After stringent quality control, a total of 12,679 cells and 24,988 genes were retained for analysis (**Supplementary Figure 3**). A total of 3,000 highly variable genes were identified for downstream analysis, and the top 30 principal components were selected for dimensionality reduction and clustering. To minimize inter-sample batch effects, Harmony was applied for data integration, resulting in improved clustering consistency and stable low-dimensional distribution across samples (**Supplementary Figure 4**). Based on canonical marker genes, cells were annotated into 10 major cell types (**Figure 11A**), including CD4+ T cells (n = 3,423), CD8+ T cells (n = 1,122), NK cells (n = 334), myeloid cells (n = 420), dendritic cells (n = 887), keratinocytes (n = 5,136), basal cells (n = 900), endothelial cells (n = 198), stromal cells (n = 50), and melanocytes (n = 209). The expression patterns of representative marker genes across these cell types are shown in **Figure 11B**, further supporting the reliability of cell annotation. The relative composition of different cell types across integrated samples was shown in **Figure 11C**, indicating a structured cellular landscape composed of immune, stromal, and epithelial compartments and providing a framework for subsequent cell-type-level interpretation of the prioritized genes.

To further define the cellular distribution of the prioritized genes, their expression patterns were examined at the single-cell level using dot plots showing mean expression and the fraction of expressing cells across major cell populations in normal and psoriatic skin (**Figure 11D**). Group-wise UMAP density plots of the four genes expression were provided in **Supplementary Figure 5**. Overall, the four candidate genes showed distinct cellular preferences. *SPTSSA* showed relatively stronger expression in basal cells and keratinocytes. This pattern suggests that *SPTSSA* may be preferentially associated with a basal-like epidermal program rather than being uniformly expressed across all keratinocyte states. *CTNS* was mainly enriched in keratinocytes, with weaker additional signal in selected non-epidermal compartments, supporting a potential association with keratinocyte-related programs. In contrast, *TNFSF10* showed a more immune-related expression pattern, with relatively stronger expression in T-cell populations and endothelial cells in psoriatic samples, suggesting a relationship with inflammatory activation. *HSD17B10* exhibited a broader distribution across multiple cell types, with relatively prominent expression in myeloid cells, dendritic cells, and T cells, indicating a potential association with immune metabolism and inflammatory regulation.

Taken together, these single-cell results indicate that *SPTSSA* and *CTNS* are more closely associated with epidermal cell programs, whereas *TNFSF10* and *HSD17B10* appear to be more strongly linked to immune-inflammatory cell populations. This integrated single-cell landscape therefore provides both the cellular context and the cell-type-specific support for the prioritized biomarkers identified from the bulk transcriptomic analyses.

### 3.11 Real-time PCR provided preliminary tissue-level validation and highlighted gene-specific heterogeneity

**Figure 12:**
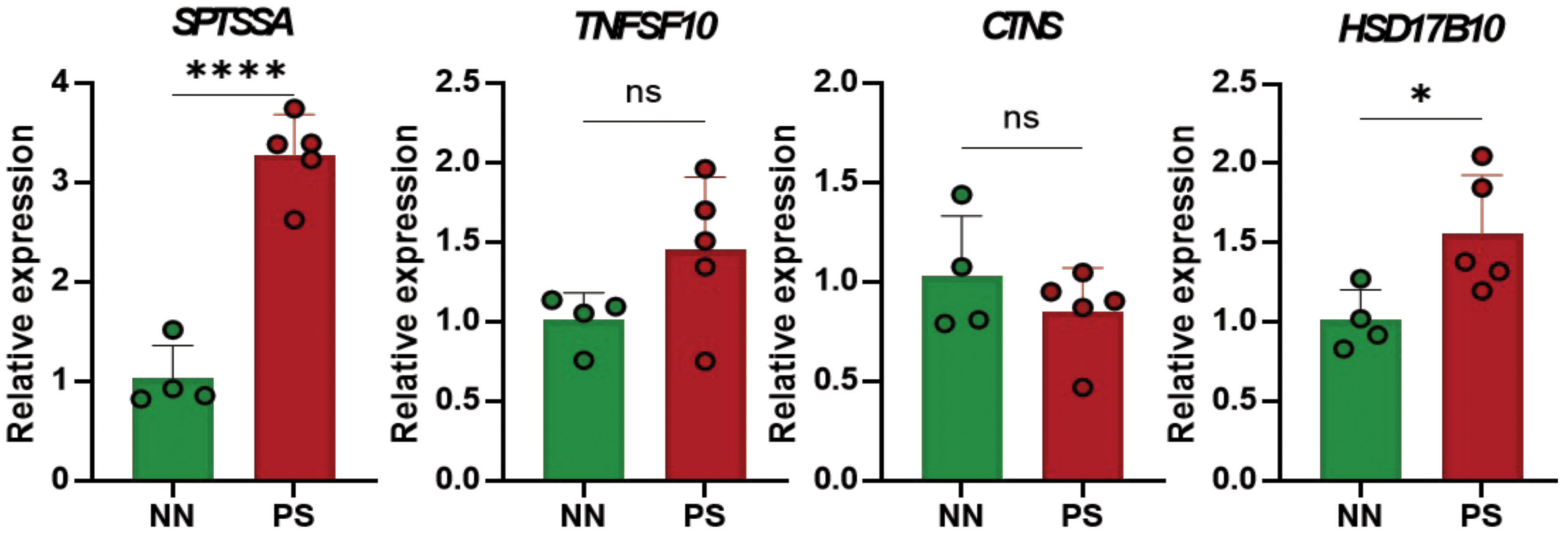
Tissue-level RT-qPCR validation of prioritized biomarkers. Relative mRNA expression of *SPTSSA*, *TNFSF10*, *CTNS*, and *HSD17B10* in psoriatic lesional skin (PS, n = 5) and healthy control skin (NN, n = 4). Each point represents one biological sample; each sample was measured in technical duplicate wells. Data are shown as individual points with summary bars. *P < 0.05, ****P < 0.0001; ns, not significant.

To assess the translational relevance of the computationally prioritized genes, real-time PCR was performed in five psoriatic lesional skin tissues and four healthy skin controls (**Figure 12**). Among the four prioritized genes emphasized in the downstream analyses, *SPTSSA* showed marked upregulation in psoriatic lesions, and *HSD17B10* was also significantly increased. *CTNS* and *TNFSF10* did not show statistically significant differences between the two groups. An exploratory analysis of *CERK* showed an opposite tissue-level trend relative to the public-dataset discovery signal (**Supplementary Figure 6**); accordingly, *CERK* is interpreted here as a mechanistically informative but directionally unresolved candidate rather than as a core tissue-validated biomarker. Taken together, these tissue-level data provide preliminary support for the translational relevance of the prioritized signature while also highlighting gene-specific heterogeneity.

## 4 Discussion

The present study identifies a palmitoylation-associated molecular signature in psoriasis through integrated analyses of bulk transcriptomic datasets, machine-learning prioritization, external validation, immune microenvironment profiling, single-cell contextualization, and preliminary tissue-level validation. Importantly, our findings should be interpreted as identifying palmitoylation-associated rather than definitively palmitoylated biomarkers or causal mechanistic involvement. This is because the current study was based primarily on transcriptomic integration, correlative analyses, and broad gene-universe annotation, without direct biochemical confirmation of protein palmitoylation. Nevertheless, the resulting signature showed biological coherence, as it was consistently associated with pathways already implicated in psoriasis, including keratinocyte activation, inflammatory immune remodeling, and sphingolipid dysregulation (1–3). Accordingly, our findings should be viewed as a biologically plausible and hypothesis-generating framework for subsequent mechanistic investigation, rather than as direct mechanistic proof.

A major strength of the study is that the identified genes are not merely statistically selected markers but are biologically anchored in lipid and barrier biology. The enrichment of ceramide and sphingolipid metabolic processes among Palm-DEGs, together with the prioritization of *SPTSSA* and *CERK*, suggests that palmitoylation-associated transcriptional alterations in psoriasis may intersect with epidermal lipid regulation. This interpretation is consistent with prior evidence showing altered ceramide synthesis, lesion-specific barrier-lipid remodeling, and stratum-corneum ceramide abnormalities in psoriasis (9, 10, 12, 13). In that context, *SPTSSA* is particularly notable because it encodes a regulatory small subunit of serine palmitoyltransferase, the enzyme that catalyzes the first and rate-limiting step in *de novo* sphingolipid biosynthesis (25–27).

Among the prioritized genes, *SPTSSA* emerged as the most biologically cohesive and tissue-supported candidate in our analysis. It showed stable cross-cohort diagnostic performance, the strongest correlation with keratinocyte markers, a close association with ceramide/sphingolipid metabolic pathways and AHR-associated signaling, and robust upregulation in psoriatic lesional tissues. These features suggest that *SPTSSA* may represent a plausible link between epidermal differentiation programs, lipid homeostasis, and inflammatory amplification in psoriasis. However, this interpretation should still be regarded as association-based rather than as direct mechanistic proof. The plausibility of this interpretation is supported by prior studies showing that AHR signaling can stimulate sphingolipid programs and that tapinarof, an AHR agonist, exerts therapeutic effects in psoriasis partly through barrier-restorative and immunomodulatory mechanisms (19–21).

*HSD17B10* also showed consistent lesional upregulation by RT-qPCR, supporting its prioritization as a translationally relevant candidate. By contrast, *CTNS* and *TNFSF10* remained strongly supported by public-dataset validation but did not reach statistical significance in the small tissue cohort. These genes should therefore be interpreted as promising but still provisional tissue-level candidates. Additional lesion/non-lesion paired cohorts and protein-level validation will be needed to clarify whether the absence of significance reflects biological heterogeneity, limited sample size, or more compartment-specific regulation.

*TNFSF10* and *CTNS* appear to contribute complementary but non-identical dimensions to the signature. TNFSF10/TRAIL has been detected in inflammatory dendritic-cell programs in psoriatic lesions and has been associated with keratinocyte susceptibility to inflammatory injury (28–30). Consistently, in our data, *TNFSF10* showed a more immune-related expression pattern and retained partial associations with macrophage and dendritic-cell markers, supporting the interpretation that the palmitoylation-associated signature is not confined to epidermal cells. By contrast, *CTNS* showed predominantly epidermal distribution, with the strongest expression in keratinocytes and weaker additional signal in selected non-epidermal compartments, suggesting a closer association with keratinocyte-related programs than with immune-inflammatory cell states. This aligns with current single-cell studies highlighting the pathogenic roles of both epidermal dysfunction and innate immune activation in psoriasis (6, 8, 31).

The generally concordant expression trends and ROC performance across GSE14905, GSE13355, and GSE121212 support a degree of cross-cohort robustness for the four prioritized genes. At the same time, these results should be interpreted with appropriate caution, because public transcriptomic cohorts may differ in platform characteristics, cohort composition, lesion heterogeneity, tissue cellularity, and unavailable clinical covariates such as treatment status or disease severity. Accordingly, the cross-cohort validation in the present study supports relative reproducibility rather than definitive clinical generalizability.

The immune microenvironment analyses further reinforce this model. High-score samples displayed enrichment of macrophages M0/M1 and activated dendritic cells together with strong inflammatory pathway activation and increased immune checkpoint expression. In psoriasis, such coexistence of immune activation and inhibitory counter-regulation is biologically plausible and should not be interpreted as a classical tumor-like immune-escape program. Rather, it may reflect a highly activated inflammatory state in which counter-regulatory mechanisms are induced in parallel. The positive correlations of the stable genes, especially *SPTSSA*, with CTLA4, TIGIT, and CD274 indicate an association with a broader immunologic state, rather than proving that these genes directly regulate immune checkpoint pathways.

*CERK* deserves special attention even though its cross-cohort diagnostic performance was weaker than that of the other stable genes. CERK catalyzes the phosphorylation of ceramide to ceramide-1-phosphate and has documented pro-inflammatory functions in TNF-α-stimulated human monocytes (32). Interestingly, our tissue RT-qPCR showed lower *CERK* expression in psoriatic lesions than in healthy controls, in contrast to its inclusion in the disease-upregulated computational signature. This discrepancy most likely reflects context dependence, sampling differences, small tissue-cohort size, or cell-composition effects. Accordingly, *CERK* is best interpreted at present as a mechanistically informative candidate rather than a stable tissue-level diagnostic biomarker.

Taken together, our findings support an integrated framework in which palmitoylation-associated transcriptional alterations are associated with sphingolipid metabolic dysregulation, which may contribute to epidermal barrier perturbation and subsequently amplify inflammatory circuits in psoriasis. In this model, epidermal-associated candidates such as *SPTSSA* and *CTNS* may link barrier–lipid programs with keratinocyte dysfunction, whereas *TNFSF10* and the more broadly distributed *HSD17B10* may reflect complementary immune-inflammatory components of the same pathogenic continuum. This integrated perspective helps place the identified genes within a broader cascade of palmitoylation-associated biology, lipid dysregulation, barrier disturbance, and inflammatory remodeling, while remaining association-based rather than directly mechanistic.

This study has several limitations. First, the broad palmitoylation-associated gene universe was derived primarily from GeneCards, which may include genes indirectly rather than directly linked to palmitoylation. Thus, the PRG list used here should be regarded as an association-level resource rather than as a curated set of experimentally confirmed palmitoylated proteins. Future work should therefore integrate curated resources such as SwissPalm/SwissPalm2 and separate broad-association genes from high-confidence palmitoylation-supported candidates. Second, the initial hub-gene screening framework focused on upregulated DEGs in order to prioritize disease-activated candidates with greater translational plausibility; however, this strategy may have reduced sensitivity for biologically relevant downregulated genes, including genes with potential homeostatic or protective roles. Third, transcriptomic and correlation-based associations do not by themselves prove direct protein palmitoylation, altered enzymatic activity, or causal mechanistic involvement in sphingolipid remodeling or immune regulation. Fourth, the tissue-validation cohort was small and included only psoriatic lesions and healthy controls; paired non-lesional samples and protein-level assays would substantially strengthen the conclusions. Fifth, although candidate compounds were identified by CTD-based association mining, these findings are hypothesis-generating and should not be interpreted as evidence of therapeutic efficacy without further experimental validation.

Overall, our results support a model in which palmitoylation-associated biology in psoriasis is linked to the convergence of keratinocyte lipid/barrier programs, sphingolipid metabolism, and inflammatory immune remodeling. This framework provides a more biologically integrated perspective than biomarker discovery alone and offers a rational basis for subsequent translational studies focused on *SPTSSA*-centered barrier metabolism and related immune-inflammatory pathways.

## 5 Conclusion

In summary, we identified a palmitoylation-associated molecular signature in psoriasis and prioritized *SPTSSA*, *TNFSF10*, *CTNS*, and *HSD17B10* as relatively stable computational candidate biomarkers, with *SPTSSA* and *HSD17B10* receiving additional support from tissue-level RT-qPCR and *CERK* retained as a mechanistically informative candidate. The signature was linked to sphingolipid metabolism, keratinocyte dysfunction, myeloid and lymphoid immune remodeling, and coordinated immune-checkpoint induction. These findings provide a clinically relevant and biologically coherent framework for future lesion/non-lesion tissue validation, protein-level verification, and mechanism-oriented studies in psoriasis.

## Data Availability

The datasets presented in this study can be found in online repositories. The names of the repository/repositories and accession number(s) can be found in the article/**Supplementary Material**.
