# Supplementary material for: Integrated transcriptomic and tissue-validation analyses identify palmitoylation-associated biomarkers linked to sphingolipid metabolism and immune remodeling in psoriasis
Source: Front Immunol. 2026 Jul 21;17:1856647. doi: 10.3389/fimmu.2026.1856647 (PMC13433182; doi:10.3389/fimmu.2026.1856647)
Supplement: Supplementary file 1 [file Table4.docx]

Supplementary Material

# 1 Supplementary Figures

**Supplementary Figure S1. Cross-cohort validation of candidate hub genes.** (A) Expression of other four candidate genes in GSE14905. (B) Expression of other four candidate genes in GSE13355. (C) Expression of other four candidate genes in GSE121212.

**Supplementary Figure S2. MCPcounter-based immune microenvironment features associated with the palmitoylation-related score.** (A) Stacked bar plot showing the relative proportions of different cell populations in each sample. (B) Spearman correlation matrix of the indicated cell populations. (C) Violin plots showing the abundance differences of each cell population between the low- and high-score groups. (D) Summary violin plot of cell abundance in the two groups. (E) Radar plot showing the relative abundance patterns of different cell populations in the low- and high-score groups.

**Supplementary Figure S3. Quality control of single-cell RNA-seq data.** (A) Violin plots showing single-cell filtering metrics. (B) Scatter plots for single-cell quality control.

**Supplementary Figure S4. Dimensionality reduction and clustering of the single-cell dataset.** (A) Top 3,000 highly variable genes. (B) PCA plot. (C) tSNE plot colored by sample batch. (D) UMAP plot colored by sample batch. (E) tSNE plot showing cell clusters at resolution 1. (F) UMAP plot showing cell clusters at resolution 1.

**Supplementary Figure S5.** **Group-wise UMAP density plot of the prioritized biomarkers expression.** (A) Density distribution of SPTSSA-associated expression signal across the UMAP embedding in normal and disease samples. (B) Density distribution of TNFSF10-associated expression signal across the UMAP embedding in normal and disease samples. (C) Density distribution of CTNS-associated expression signal across the UMAP embedding in normal and disease samples. (D) Density distribution of HSD17B10-associated expression signal across the UMAP embedding in normal and disease samples

**Supplementary Figure S6. Tissue-level RT-qPCR validation of *CERK*.** Relative mRNA expression of *CERK* in psoriatic lesional skin (PS, n = 5) and healthy control skin (NN, n = 4). Each point represents one biological sample; each sample was measured in technical duplicate wells. Data are shown as individual points with summary bars. ***P* < 0.01.

# 2 Supplementary Tables

**2.1 Supplementary Table 1-GeneCards-palmitoylation related genes**

**2.2 Supplementary Table 2-primers**

**2.3 Supplementary Table 3-ssgseaScore**
